# Severe sepsis bundles

**DOI:** 10.4103/0972-5229.63028

**Published:** 2010

**Authors:** Parvez Khan, J. V. Divatia

**Affiliations:** **From:** Department of Anaesthesia, Critical Care and Pain, Tata Memorial Hospital, Mumbai, India

**Keywords:** Severe Sepsis, Surviving Sepsis Campaign, Sepsis Care Bundles

## Abstract

Sepsis is a complex syndrome with its wide spectrum of severity, and is one of the most common causes of death in Critical Care Units. The Surviving Sepsis campaign launched in 2004, is aimed at improving diagnosis, management and survival of patients with sepsis. Care bundles are a group of best evidence based interventions which when instituted together, gives maximum outcome benefit. Care Bundles are simple, uniform and have universal practical applicability. Surviving Sepsis campaign guidelines in 2008 incorporated two sepsis care bundles. The Resuscitation bundle includes seven key interventions to be achieved in 6-h while four interventions have to be completed within 24-h in the Management bundle. Compliance with a bundle implies achieving all the specified goals in that bundle. Limitations to care bundles include the quality of the evidence on which they are based, and that the relative contributions of each element of the bundle are not known. Several observational studies support the hypothesis that sepsis care bundles have an important role in improving outcomes from sepsis. Critical Care Units should develop management strategies to ensure compliance with the sepsis bundles in order to decrease hospital mortality due to severe sepsis.

## Introduction

Sepsis is well documented as a complex syndrome, at times with severe magnitude, which comprises a wide range of adverse clinical conditions manifesting as negative fall-outs of the body's systemic response to an infection. Severe sepsis is accompanied by single or multiple organ dysfunction or failure, eventually leading to death.[1] Owing to its aggressive, multifactorial nature, sepsis is a rapid killer affecting millions of human population worldwide and studies have recorded that the mortality rate due to sepsis has been as alarming as one in four (or even more), with its incidence increasing unabated. Therefore, any disease process that needs development of effective therapies relies on the ability to clearly define the disease and identify the targeted population of patients who will specifically benefit from that intervention. The evolution of universally accepted definitions would, therefore, be a vital first step in helping the medical community in the course of its effective sepsis management.[1] Faced with complexity and variability of sepsis, with its potential to inflict deadly consequences upon the people afflicted the definition as evolved now as a result of deliberations over a decade conforms to the present diagnostic criteria arrived at the consensus conference in 2003.[2]

## Surviving Sepsis Campaign

Advances in our understanding of heterogeneity of sepsis pathogenesis have made it clear that any single therapeutic intervention is probably too simple, although some interventions may target more general pathways and, therefore, be globally beneficial. Similar to polytrauma, acute myocardial infarction or stroke, the speed and appropriateness of therapy administered in the initial hours after onset of severe sepsis are likely to influence outcome. The rapid diagnosis and effective management of sepsis is critical to successful treatment.[3] The surviving sepsis campaign (SSC) is aimed at improving the diagnosis, management, and survival of patients afflicted with sepsis, which is accomplished by addressing the challenges associated with it. In 2004, three phases of SSC were proposed.[4]

Phase 1: Six-point action plan to effectively reduce globally, mortality rate by 25% by the end of 2009.

Phase 2: Creating appropriate guidelines for management (Sepsis guidelines).

Phase 3: Translating guidelines into clinical practice. Campaign leaders partnered with the Institute for Healthcare Improvement (IHI) to develop two sepsis bundles and to create a database-centered change measurement process. These bundles and the supporting evidence-based guidelines form the basis for best practice recommendations.

Duly recognizing that implementation of the guidelines in clinical practice presents a significant challenge, the SSC set out to develop and evaluate a multifaceted model incorporating necessary changes to bedside practice to be consistent with the recently published management guidelines.[5] Although care bundle approaches have been practiced across a variety of clinical indications, particularly in cardiology, for more than two decades, it is only in this decade that their actual application in the sepsis management has evolved. The very high level of ever-increasing interest in sepsis care bundles demands that makes it all the more important and appropriate to understand their key components, not just their successes but also their limitations.[4]

## Care Bundles

Bundles are a group of “therapies” built around the best evidence-based guidelines, which, when implemented together, produce greater benefit in terms of outcome than the individual therapeutic interventions. The conception of care bundles has been proposed based on the holistic principle that the whole is greater than the sum of its parts. On the basis of this therapeutic approach, the IHI and the Centers for Medicare and Medicaid Services recently proposed instituting “all or none” performance measures.[6,7]

A bundle process that combines the best of medical science and improvement science is developed in the following methods.

1. Identifying a set of four-to six-evidence-based interventions that apply to a cohort of patients with a common disease or a common location.
2. Developing the will in the providers to deliver the interventions every time they are presented with such indications.
3. Measuring compliance with practice guidelines as “all” or “nothing.”
4. Redesigning the delivery system to ensure that the interventions in the bundle are delivered.
5. Measuring the related outcomes to ascertain the effects of the changes in the delivery system.

The sepsis bundles were developed in just an identical manner, based on the experience of the ventilator bundle.[8]

## Need for Bundles

Bundle effectiveness is resulted due to the excellence of the supporting evidence and its consistent comprehensive execution, with the impact levels being greater by performing all elements together rather than in isolation, that is by performing any individual component. Although bundle elements are relatively not new, and have a strong clinical base, because of lack of uniformity in performance in normal practice, treatment is unreliable and subjective driven on occasion by individual idiosyncrasies. Bundles play a useful role to help remove the constraints imposed by these deviations and variations by means of constructing the elements into packages that must be implemented in strict compliance for every patient, at each and every single time to ensure uniformity as well as universality. It is this simplicity and inherent strength that have enhanced the attractiveness and practical applicability of this approach.[4,9]

## Sepsis Care Bundles

There are two severe sepsis bundles: sepsis resuscitation bundle, and sepsis management bundle, which will be discussed in detail. Each bundle articulates objectives to be accomplished within specific timeframes. The bundles have been developed based on the 2008 Surviving Sepsis Campaign Guidelines for the Management of Severe Sepsis and Septic Shock. The Guidelines incorporated an evidence-based review of the literature and assessment through rankings according to the strength of each recommendation.[10]

## Sepsis Resuscitation Bundle

The resuscitation bundle is a combination of evidence-based objectives that must be completed within 6 h for patients presenting with severe sepsis, septic shock, and/or lactate >4 mmol/L (36 mg/dL).

For patients with severe sepsis, as many as seven bundle elements must be accomplished within the first 6 h of presentation.

- Measure serum lactate
- Obtain blood cultures prior to antibiotic administration
- Broad-spectrum antibiotic within 3 h of ED admission and within 1 h of non-ED admission (improved time to administration)
- Treat hypotension and/or elevated lactate with fluids
- Administer vasopressors for hypotension not responding to initial fluid resuscitation to maintain mean arterial pressure (MAP) >65 mmHg.
  In the event of persistent hypertension despite fluid resuscitation (septic shock) and/or lactate >4 mmol/L, maintain adequate central venous pressure (CVP) and central venous oxygen saturation
- Achieve a CVP of >8 mmHg
- Achieve central venous oxygen saturation (ScvO_2_) >70% or mixed venous oxygen saturation (SvO_2_) >65%.

## Sepsis Management Bundle

This consists of evidence-based objectives that must be completed within 24 h for patients with severe sepsis, septic shock, and/or lactate >4 mmol/L (36 mg/dL). For patients with severe sepsis, as many as four bundle elements must be accomplished within the first 24 h of presentation. The objective should be to adhere to the norms of performing all the indicated tasks as detailed below each and every time within the first 24 h of presentation.[7]

- Administer low-dose steroids for septic shock in accordance with a standardized ICU policy. If not administered, then document why the patient did not qualify for low-dose steroids based on the standardized protocol.
- Administer recombinant human activated protein C (rhAPC) in accordance with a standardized ICU policy. If not administered, then document why the patient did not qualify for rhAPC.
- Maintain glucose control lower than limit of normal, but < 180 mg/dL(10 mmol/L).
- Maintain a median inspiratory plateau pressure (IPP) < 30 cm H_2_ O for patients under mechanical ventilation.

## Bundle Targets and SSC Performance Improvement Initiative

Gao *et al*,[11] carried out the first study to demonstrate the impact of compliance after adopting the SSC 6-h and 24-h sepsis bundles on hospital mortality in patients with severe sepsis. They found the rate of compliance with all the aspects of the 6-h and 24-h sepsis bundles to be 52% and 30%, respectively. Noncompliance with the 6-h sepsis bundle was associated with a more than twofold increase in hospital mortality. There was a 76% increase in risk for hospital death if the 24-h bundle targets were not achieved. The compliant and the noncompliant groups were comparable without much of significant difference in their characteristics and severity of sepsis. The NNT to save one life was determined to be approximately 4. Although the study suffered from constraints of being merely observational and had other limitations including a small sample size and not taking account of evaluating other risk factors for death (organ failure scores) or accounting for patients who did not require critical care admission, it was one of the first to document a clinical benefit associated with Sepsis Bundle implementation.

Phase 3 of the SSC seeks to facilitate operationalizing of the guidelines to create a global golden standard of care for sepsis management.[4,12] The SSC performance improvement initiative was launched in multiple sites worldwide on a voluntary basis to measure changes in the rates at which the sites achieved the targets of the guideline bundles and to assess the impact of compliance with the program on hospital mortality. Levy *et al*,[12] recently analyzed data compiled from 15,022 subjects at 165 sites, through eight quarters over a period of 2 years. Results showed that compliance with the initial 6-h bundle targets increased linearly from 10.9% of subjects in the first-site quarter to 31.3% by the end of 2 years of the campaign program, achieving improved compliance of statistical significance by the second quarter itself. Compliance for the entire 24-h management bundle started higher with 18.4% but did not achieve statistical significance. The hospital mortality in both the unadjusted and adjusted models showed greater decrease, provided that a site stayed in the campaign for a longer duration, resulting in an adjusted absolute drop of 0.8% per quarter and 5.4% over the first 2 years (95% CI, 2.5–8.4). Overall, the relationship between achievements of targets for bundles and mortality was significant and encouraging.[12] After adjustment for baseline characteristics, administration of broad-spectrum antibiotics (OR, 0.86; 95%, CI 0.79–0.93), obtaining blood cultures before their initiation (OR, 0.76; 95% CI, 0.70–0.83), and maintaining blood glucose control (OR, 0.67; 95% CI, 0.62–0.71) were all associated with lower hospital mortality. Measuring lactate was, however, not associated with improved outcome. The administration of drotrecogin alfa in the first 24 h was associated with improved survival in those with shock (OR, 0.81; 95% CI, 0.68–0.96). For those who required mechanical ventilation, achieving plateau pressure control was associated with improved outcome (OR, 0.70; 95% CI, 0.62–0.78). In those with septic shock, there was no association between mortality and the use of low-dose steroids, the ability to achieve a central venous pressure ≥8 mmHg, or ScvO_2_ ≥70%.

In order to assess the association between outcome and the utilization of component therapies (protocol-based care) in the studies of sepsis bundles, a meta-analysis[13] of one randomized controlled trial[14] and seven trials with historic controls[15–21] was performed. These studies are summarized in Table 1. Bundle use was associated with consistent and significant improvement in survival, and the antibiotic administration with timing and appropriateness had maximum outcome benefit. The use of other bundle components varied heterogeneously across studies, with their impact on survival being inconclusive and not definitive. Resuscitation fluid volumes, percentage of patients receiving vasopressors, administration of PRBCs and inotropes to obtain an ScvO_2_ of ≥70% were not consistent in registering changes with bundled care over the eight studies. Insulin therapy and lung protective strategies were insufficiently reported and, therefore, not analyzed. However, other factors may have also made their respective contributions independent of component therapies. A limitation of this meta-analysis is due to the lack of methodologic rigor in the studies analyzed.

**Table 1:** Study design and bundle care treatment and mortality end points, Adapted from Barochia *et al*.[13]

Study (reference)	Rivers *et al*,[14]	Trzeciak *et al*,[15]	Kortgen *et al*,[16]	Shapiro *et al*,[17]	Micek *et al*,[18]	Nguyen *et al*,[19]	Jones *et al*,[20]	El Solh *et al*.[21]
Year published	2001	2006	2006	2006	2006	2007	2007	2008
Study design	Prospective, randomized	Before-after	Before-after	Before-after	Before-after	Before-after	Before-after	Before-after
Setting	ED	ED/ICU	ICU	ED/ICU	ED/ICU	ED/ICU	ED	ED/ICU
Aids to facilitate protocol cared	Yes	No	No	Yes	Yes	Yes	Yes	Yes
Initial treatment time	0–6 h	Time in ED	0–6 h	0–6 h	Time in ED	Time in ED	0–6 h	0–6 h
Protocol treatments								
Antibiotics	No	No	Yes	Yes	Yes	Yes	Yes	Yes
EGDT	Yes	Yes	Yes	Yes	Yes	Yes	Yes	Yes
Corticosteroids	No	No	Yes	Yes	Yes	Yes	No	Yes
rhAPC	No	No	Yes	Yes	Yes	No	No	Yes
Low tidal volume	No	No	Yes	Yes	No	No	No	No
Intensive insulin	No	No	Yes	Yes	No	No	No	Yes
Mortality end point	In hospital	In hospital	28 days	28 days	28 days	In hospital	In hospital	28 days

All the trials were unblended.

ICU, intensive care unit; ED, emergency department; EGDT, early goal-directed therapy; rhAPC, recombinant human activated protein C.

Importantly, six of the trials described education or treatment aids to improve bundle utilization. A large multicenter Spanish trial[22] showed the impact of education on target achievement with sepsis bundle. The educational program consisted of training physicians and nursing staff from the emergency department, wards, and ICU in imparting knowledge regarding the definition, recognition, and treatment of severe sepsis and septic shock as outlined in the SSC guidelines. A national educational effort to promote bundles of care was associated with improved guideline compliance and lower hospital mortality. Compliance with the sepsis resuscitation bundle returned to baseline after 1 year, but compliance with the sepsis management bundle and mortality remained stable with respect to the post-intervention period.

In India, Raymond and his colleagues from Chennai performed an observational study (unpublished observations, personal communication) to assess the adherence to and compliance with the SSC bundles in their multidisciplinary ICU, and the impact of compliance to the bundles on outcome. In the study, 277 patients with severe sepsis or septic shock were investigated. Mortality rate of the study population was 31.4%. All the objectives were achieved in 69 patients (25%). The mortality in patients achieving all the objectives was significantly lower compared to the patient population in whom all the objectives were not accomplished (21.7% vs. 34.6%, *P* = 0.01). ICU-free and ventilator-free days were also significantly lower showing thereby the significant improvement in complying with the SSC bundle scheme.

Overall, 148 ICUs from 16 countries across Asia recently participated in the Management of Sepsis in Asia's Intensive Care Units (MOSAICS) study. This was a prospective multicenter, observational study performed to document the compliance of Asian ICUs with the recommendations of the SSC resuscitation and management bundles. Secondary objectives include documenting the epidemiology and outcomes of severe sepsis in Asian ICUs and to evaluate whether compliance of Asian ICUs with the recommendations within the SSC resuscitation and management bundles lead to improved outcomes. Totally, 1285 patients were enrolled, among whom 162 patients were from 17 Indian ICUs. All the consecutive patients with severe sepsis or septic shock undergoing therapy in the participating ICUs in the month of July 2009 were recruited. Patients below 21 years of age, those admitted from other ICUs, and those previously admitted to the ICU with sepsis were excluded. Primary outcome that was the focus of this study was all-cause mortality. The results for the outcome are awaited.

## Limitations of Bundled care

Care bundles differ from standard care pathways in the way that compliance is measured and accordingly rated i.e., only if all elements of the bundle are applied, will then the healthcare team receive a “pass” The team fails even though they achieve all the targets excepting even one (all or none). The experience so far is that the management bundle is the least controversial and easiest to introduce and find acceptance. The resuscitation bundle is derived from the study by Rivers based on early-goals directed therapy[14] which has its own lacunae. The SSC guidelines were developed by consensus, and some of the end-points represent a pragmatic compromise which eventually will evolve over time with stronger evidence base in future.[12] The beneficial effect of the guidelines on patients outcomes is subject to scrutiny and remains unproven, and the primary evidence obtained so far lacks substance and quality to promote the guidelines as a global standard of care for universal applicability. However, the most conservative conclusion drawn from this recent analysis is that doing so is unlikely to cause any harm.[23]

## Conclusions

Overall, we believe that the SSC provides an opportunity for the critical care community to further bridge the gap between evidence and application. It is the single most important initiative to have occurred in modern critical care. For the first time, standards of care on the intensive care unit are being defined and, therefore, intensivists must respond to them. A Questionnaire that evaluated ventilator-associated pneumonia recommendations among experts found an overall 37% no-adherence rate. Compliance was not positively associated with the weight of evidence, with 35% of clinicians disagreeing on conclusions of clinical trials.[24] More credible process measurements are essential to induce positive changes in the intervention and treatment care. Measurements for improvement should be simple and easily adaptable. Data of this study support the hypothesis that sepsis care bundles have an important role in future septic infection management. As a consequence, any effort undertaken for decreasing the hospital mortality due to severe sepsis should focus on increasing and encouraging compliance with these evidence-based interventions in the case of patients.
